# WiFi Fingerprinting Indoor Localization Based on Dynamic Mode Decomposition Feature Selection with Hidden Markov Model

**DOI:** 10.3390/s21206778

**Published:** 2021-10-13

**Authors:** Oluwaseyi Paul Babalola, Vipin Balyan

**Affiliations:** Department of Electrical, Electronics and Computer Science Engineering, Faculty of Engineering and the Built Environment, Cape Peninsula University of Technology, Bellville 7537, South Africa; balyanv@cput.ac.za

**Keywords:** dynamic mode decomposition (DMD), hidden Markov model (HMM), machine learning, RSSI, WiFi fingerprinting

## Abstract

Over the years, WiFi received signal strength indicator (RSSI) measurements have been widely implemented for determining the location of a user’s position in an indoor environment, where the GPS signal might not be received. This method utilizes a huge RSSI dataset collected from numerous access points (APs). The WiFi RSSI measurements are nonlinear with distance and are largely influenced by interference in the indoor environment. Therefore, machine learning (ML) techniques such as a hidden Markov model (HMM) are generally utilized to efficiently identify a trend of RSSI values, which corresponds to locations around a region of interest. Similar to other ML tools, the performance and computing cost of the HMM are dependent on the feature dimension since a large quantity of RSSI measurements are required for the learning process. Hence, this article introduces a feature extraction method based on dynamic mode decomposition (DMD) for the HMM to effectively model WiFi fingerprint indoor localization. The DMD is adopted since it decomposes RSSIs to meaningful spatial and temporal forms over a given time. Here, the mode forms are analytically reconstructed to produce low-dimensional feature vectors, which are used with the HMM. The localization performance of the proposed HMM-DMD is compared with other well-known ML algorithms for WiFi fingerprinting localization using simulations. The results show that the HMM-DMD algorithm yields a significant localization performance improvement, accuracy, and reasonable processing time in comparison with the state-of-the-art algorithms.

## 1. Introduction

Indoor localization is critical for a variety of location-aware services, including health care monitoring, tracking, mobile resource management, and fraud detection. WiFi has received growing interest among other indoor localization systems, such as radio-frequency identification (RFID), Bluetooth, ultrasound, etc. This is due to the widespread implementation of wireless local area networks (WLANs) in indoor environments, and the ubiquity of mobile devices that are compatible with WiFi systems, thus providing a relatively low-cost method of user monitoring in the indoor environment. The WiFi fingerprinting localization technique is commonly implemented for location approximation since it does not require historical information of wireless access point (AP) distribution and does not necessitate computing a receiver’s angle. The technique utilizes received signal strength indicator (RSSI) measurements of accessible APs to predict the position of a user device in areas where the Global Positioning System (GPS) is insufficient, such as the indoor environment.

The WiFi RSSI fingerprinting process has two stages: offline or training and online monitoring or testing. In the training stage, multidimensional vectors of RSSI values (fingerprints), are collected from various APs in a range and linked to identified locations. These measurements are used to develop a database (radio map) that spans the area of interest. During the online stage, a current user device obtains an RSSI vector at an unidentified location, which is compared to the stored RSSI vector in the fingerprint. The stored information in the WiFi RSSI database is highly inconsistent due to the varying visibility of the APs. Factors such as reflections, multipath interference, and changes in the environment or device configuration affect the received signal of the APs and reduce the accuracy of the WiFi RSSI fingerprinting-based localization system, especially in the indoor environment. Note that the WiFi RSSI fingerprint database has fewer degrees of freedom compared to the dimensions of the database since the WiFi RSSI measurements are nonlinear with distance traveled by the signal, showing a spatial correlation of RSSI data. Furthermore, the fingerprint database has temporal correlation, considering the similarity that exists between RSSI values obtained by an AP at different times in a fixed position. Therefore, deterministic methods such as *k*-nearest neighbor (KNN) [1] and probabilistic Bayesian methods such as Horus [2], Kalman and particle filters [3,4], and hidden Markov models (HMMs) [5,6] are generally used as location estimation algorithms to obtain an enhanced system accuracy.

As opposed to the deterministic approach, the probabilistic methods achieve greater location accuracy since they solve uncertainty problems [2]. Specifically, the HMM is a versatile probabilistic technique for sequential data modeling that has applications in natural language processing, acoustic monitoring, speech recognition, and other fields. As of late, the HMM has been used for indoor localization due to its high efficiency and accuracy compared to other Bayesian-based methods. Considering the spatial–temporal relationship of the WiFi signals, temporal autocorrelation was utilized to properly estimate parameters of the HMM in [2]. The temporal autocorrelation was also used in [7] to develop an indoor localization model in the offline stage and to estimate a user’s location in the online stage.

In addition to improving accuracy of the system, other concerns such as computational complexity, device power, and storage must be considered for resource-constrained user devices [8]. These factors are usually determined by the number of APs in a range, that is, the measured RSSI feature dimension. Generally, the dimensionality of the features is huge in the indoor environment, making real-time implementations unrealistic. Additionally, since APs cannot cover large areas, some of the entities in the RSSI database are typically empty, which reduces the accuracy of the HMM. Hence, extracting the required set of features from the massive RSSI vector in the database without leaving out important spatial and temporal information is a major challenge.

In the literature, several feature extraction methods have been considered for data dimensionality reduction. In [9], a small portion of recognizable APs was intuitively selected for the localization system to reduce the cost of computation, power consumption, and storage capacity of the devices. Nevertheless, the approach ignores APs that may contain useful information, thus affecting the system’s performance. An error analysis-based heuristic AP selection algorithm with the aim of maintaining highly contributing APs while reducing redundant AP interference was proposed in [10]. Moreover, it is challenging to explicitly model the interference due to its variation. Thus, removing redundant APs can incorrectly remove valuable information, affecting the accuracy of localization. The traditional deep neural network (DNN) technique [11] has been utilized in the WiFi RSSI localization system. The DNN has structures that are capable of extracting complex features from RSSI observations and producing high accuracy. However, feature extraction is performed within the hidden neural network layers, which are linked to the most recent localization node. As a result, the technique is not suitable for the objective of independently obtaining reduced dimensional feature vectors.

Discriminant analysis such as linear discriminant analysis (LDA) or Fisher’s discriminant analysis (FDA) [12,13] and principal component analysis (PCA) [14,15,16] are among the well-known feature dimension reduction techniques for RSSI fingerprinting systems. In [14], a new collection of principal components (PCs) was obtained by reconstructing RSSI measurements based on the PCA.The PCA aims to reduce the mean square reconstruction error as much as possible by selecting a portion of PCs while maintaining relevant data from all of the APs for the localization model. In [15], a kernel PCA was developed to achieve a nonlinear mapping between the PCs and the user position, which shows an improvement on [14]. To reduce the impact of environmental interference, AP clusters were constructed in [16], where hierarchical PCA was used to obtain cluster RSSI features by converting the raw data to a linearly independent dimensional matrix. However, since PCA is an unsupervised learning method, it may ignore the information needed to execute classification of a state that exists in the direction of small eigenvalues (regarding the direction as noise) [17]. Therefore, ref. [18] considered the spatial–temporal WiFi RSSI data relationships and presented a combination of the FDA with PCA to extract features from the original RSSI datasets using semisupervised machine learning. Additionally, ref. [19] exploited the spatial–temporal correlations of RSS datasets to transform the fingerprint database to a low-rank matrix based on robust PCA.

In this article, dynamic mode decomposition (DMD) [20] is proposed as a feature extraction technique to be used with the HMM to reduce data dimensionality and enhance the accuracy of predicting a user’s location while reducing the computational complexity. DMD is a data-driven approach for evaluating nonlinear system behavior with large measurement dimensions and for decomposing the data into spatial and temporal modes with low dimensionality. This dimensionality reduction approach was initially used in the field of fluid flow [20]. Moreover, it is now used to extract unique features in forecasting, imaging, and other fields. As far as is known, DMD has not been implemented for the HMM in WiFi fingerprinting-based indoor localization. Hence, DMD is proposed as an effective method to deal with incompleteness in missing WiFi RSSI data and select relevant features from the huge number of RSSI measurements without losing useful information. Unlike choosing a small portion of recognizable APs, the decomposed modes are conceptually restructured to obtain the desired features, which are applicable for the HMMs. In addition, the proposed approach reduces the computational complexity for resource-constrained user devices.

The rest of this article is structured in the following manner. The WiFi fingerprinting approach for indoor localization is discussed in Section 2. A detailed description of the well-known feature extraction techniques and the proposed HMM-DMD localization algorithm is given in Section 3. Section 4 elaborates on the HMM classification method. Section 5 contains the results, interpretation, and discussion. Finally, the conclusion is stated in Section 6.

## 2. WiFi Fingerprinting Method for Indoor Localization

Consider an indoor environment where WiFi RSSI values are collected by a mobile user from all available APs in pre-defined reference points (RPs), which are uniformly assigned on grids in an area of interest. Let $A_{i},i=1,2,\ldots d$ and $R_{j},j=1,2,\ldots ,R$ be the amount of available APs and RPs, respectively. The mobile user scans WiFi signals from all the APs at each RP such that the collected RSSI values are stored as a fingerprint $F_{j}$ in a radio map. The radio map of all fingerprints $F=[F_{1},F_{2},\ldots ,F_{R}]$ at each RP with corresponding location coordinates $c_{j}=\left[x_{j},y_{j}\right]$ is given by:

(1) $$\Omega =\left[\begin{array}{cc} \left(x_{1},y_{1}\right) & F_{1}\\ \left(x_{2},y_{2}\right) & F_{2}\\ \vdots & \vdots \\ \left(x_{R},y_{R}\right) & F_{R} \end{array}\right]=\left[\begin{array}{ccccc} \left(x_{1},y_{1}\right) & f_{1,1} & f_{1,2} & \cdots & f_{1,d}\\ \left(x_{2},y_{2}\right) & f_{2,1} & f_{2,2} & \cdots & f_{2,d}\\ \vdots & \vdots & \vdots & \cdots & \vdots \\ \left(x_{R},y_{R}\right) & f_{R,1} & f_{R,2} & \cdots & f_{R,d} \end{array}\right]$$

Moreover, the user’s device obtains a new fingerprint $F_{n}$ in the online stage, such that the unknown user location ${\hat{p}}_{n}$ is estimated based on the radio map’s coordinate information. Note that the dimensionality of Ω is determined by the number of obtainable APs and RPs. The user device detects several APs in the indoor environment, increasing the dimension of Ω and the computing cost of the algorithm. Additionally, if any of the APs fail, the accuracy of the fingerprinting system may be compromised due to missing RSSI information. Thus, the Ω is important for effectively training the HMM to accurately determine the user’s location.

## 3. Feature Extraction Techniques for HMM

Feature selection methods are often based on mathematical formulation and transformations that maximize the variation among several obtained RSSI vectors in the radio map. In this section, the well-known FDA, PCA, and proposed DMD feature extraction techniques are discussed in detail.

### 3.1. Feature Extraction by FDA

FDA is a general classification approach for determining the projection that optimizes a between-class covariance matrix while limiting the within-class covariance matrix of observations. In a typical FDA, signals are remapped to a new space, resulting in the loss of significant signal features. Suppose ${\left\{\left(c_{j},F_{j}\right)\right\}}_{j=1}^{l}$ is a set of *l* annotated feature samples with *d*-dimensions, where $c_{j}\in \left\{1,2,\cdots ,R\right\}$ represents the associated label of $F_{j}$. The sample mean vector with *d*-dimensions in a class *k* is given by [21]:

(2) $$\mu_{k}=\frac{1}{l_{k}}\sum_{j:c_{j}=k}F_{j}.$$

The between-class covariance matrix $\Lambda_{b}$ is given by [21]:

(3) $$C_{b}=\sum_{k=1}^{M}l_{k}\left(\mu_{k}-\mu \right){\left(\mu_{k}-\mu \right)}^{T},$$

where μ is the overall mean, defined as:

(4) $$\mu =\frac{1}{l}\sum_{j=1}^{M}l_{j}\mu_{j}.$$

In addition, the within-class covariance matrix $\Lambda_{w}$ is determined by [21]:

(5) $$C_{w}=\sum_{k=1}^{M}\sum_{j:c_{j}=k}\left(F_{j}-\mu_{k}\right){\left(F_{j}-\mu_{k}\right)}^{T}.$$

Hence, the transformation matrix of the FDA is obtained by:

(6) $$W=\underset{T}{\mathrm{argmax}}\left[\mathrm{tr}\left(W^{T}C_{b}W{\left(W^{T}C_{w}W\right)}^{-1}\right)\right].$$

Furthermore, ref. [17] adapted Fisher’s discriminant ratio (FDR) averaging forms to calculate the feature ranking requirement for more than one class as:

(7) $$FDR\left(m\right)=\sum_{k=1}^{M}\sum_{h\neq k,h=1}^{M}\frac{|\mu_{k}^{m}-\mu_{h}^{m}{|}^{2}}{\sum_{k=1}^{M}d_{k}^{m}},$$

where *m* is the feature index, and FDR(m) is the average of all classes. To generate the reduced feature vectors from *W*, the features were ordered in descending order of FDR(m) with the *r* highest FDR(m) outputs being retained. The reduced *r*-dimensional feature vectors were created and identified for the HMMs after careful reconstruction. However, the LDA methods may be ineffective in solving the nonlinear WiFi RSSI fingerprinting localization system.

### 3.2. Feature Extraction by PCA

The fundamental idea of the PCA is to transform the measured RSSI vector, $F_{i},\;i=1,2,\ldots ,d$, into a collection of principal components (PCs), $\phi_{1},\phi_{2},\ldots ,\phi_{r}$, r<d. The PCs represent the largest amount of possible variance, which is obtained by the eigen-decomposition of all covariance matrices of the WiFi RSSI samples. Suppose θ=Fϕ represents a transformation between $F\in R^{d}$ and $\theta \in R^{r}$, where r<d, the PCA can then be described as orthogonal mappings of the RSSI vectors onto the subspace spanned by the top *r* biggest eigenvectors of the covariance matrix. In addition, it is considered that the directions relating to small eigenvalues provide minimal information.

To overcome the nonlinear localization problem, a kernel-based PCA was proposed in [14]. To make the RSSI vectors linearly independent, a nonlinear mapping was formed between the fundamental space of RSSI vectors $R^{d}$ and a low-dimensional feature space F, that is, $\zeta :R^{d}\to F$. Hence, the RSSI vectors’ covariance matrix in the spatial domain is given by [14]:

(8) $$C=\frac{1}{\upsilon }\sum_{i=1}^{\upsilon }\zeta \left(F_{i}\right)\zeta {\left(F_{i}\right)}^{T},$$

where υ is the number of samples required for training. Subsequently, the transformation matrix of the PCA can be derived by:

(9) $$W^{pca}=\underset{W}{\mathrm{argmax}}\left[\mathrm{tr}\left(W^{T}CW{\left(W^{T}W\right)}^{-1}\right)\right],$$

where tr(·) denotes a matrix trace.

### 3.3. Feature Extraction by DMD

Beyond the LDA/FDA and PCA feature extraction techniques, the DMD approach is presented in this article. DMD improves on the PCA feature extraction technique by combining the PCA spatial dimensionality reduction method with Fourier transforms of signal in time to create low rank modes, thus making it a powerful tool to be used with the HMM for indoor localization. DMD has been adopted over the years to study the dynamics of nonlinear systems based on Koopman operators [22] and can be easily evaluated using basic dynamical systems approaches.

In this article, DMD is introduced as a feature extraction method for a nonlinear WiFi fingerprinting localization system. Consider a nonlinear system with observation matrix $X=[x_{1},x_{2},\ldots ,x_{n}]$, where *n* denotes the number of measurements obtained over an equally distributed time frame. If the signal slowly evolves, it is possible to construct the *n*th RSSI measurement vector as a sum of scalar multiples of the prior n−1 observed RSSI vector and a residual vector *e* described by [20]:

(10) $$x_{n}=a_{1}x_{1}+a_{2}x_{2}+\cdots +a_{l-1}x_{n-1}+e.$$

The mapping in (10) is linear over time but does not imply linearizing the dynamics since the underlying dynamics that produce $x_{n}$ are nonlinear. To minimize the residual vector in (10), *X* is decomposed to matrices $X_{1}$ and $X_{2}$, where $X_{2}$ is a right cyclic shift of $X_{1}$, expressed as:

(11) $$X_{1}\in X=\left[x_{1},x_{2},\ldots ,x_{n-1}\right],$$

(12) $$X_{2}\in X=\left[x_{2},x_{3},\ldots ,x_{n}\right].$$

Note that $X_{1}$ and $X_{2}$ have the same column lengths *l* and overlap over time.

Let *M* be a matrix that relates $X_{1}$ and $X_{2}$, that is, $MX_{1}\approx X_{2}$ and

(13) $$M=X_{2}X_{1}^{⋆},$$

where $X_{1}^{⋆}$ is the pseudoinverse of $X_{1}$. In a WiFi indoor environment, *M* is a large *d*-dimensional matrix with eigen-decomposition being computationally expensive. Thus, a rank-reduced formulation in the form of a projection matrix $\hat{M}$ is considered:

(14) $$M=\underset{\hat{M}}{\mathrm{argmin}}\|X_{2}-X_{1}\hat{M}\|.$$

This process is discussed as follows:

Step 1: Reduce the rank of the fingerprints based on a singular value decomposition (SVD) of $X_{1}$ as:

(15) $$X_{1}=UDV^{T},$$

where $.^{T}$ is a conjugate transpose. *U* is an n×κ matrix containing the proper orthogonal modes of $X_{1}$ and orthonormal columns, *D* is a κ×κ diagonal matrix with singular values of $X_{1}$ sorted in decreasing order, and *V* is the right unitary matrix of $X_{1}$ with SVD target rank κ.

Step 2: Using the SVD, the matrix *M* in (13) can be derived based on $X_{1}^{⋆}$ as:

(16) $$M=X_{2}VD^{-1}U^{T}.$$

However, a rank-reduced projection matrix $\hat{M}$, which maps *M* onto the proper orthogonal modes is computed:

(17) $$\hat{M}=U^{T}MU=U^{T}X_{2}VD^{-1}.$$

This addresses the issue of computational complexity.

Step 3: Calculate the eigenvectors and eigenvalues from $\hat{W}$ so that

(18) $$\hat{M}\varphi =\varphi \tau ,$$

where the eigenvectors are represented by the columns of matrix φ, while the related eigenvalues are represented by the diagonal matrix τ.

Step 4: Determine the DMD modes, ϖ from φ, as:

(19) $$\varpi =U\varphi =X_{2}VD^{-1}\varphi .$$

Therefore, the modes are denoted in matrix form as:

(20) $$\varpi =\left[\begin{array}{cccc} \varpi_{1,1} & \varpi_{1,2} & \cdots & \varpi_{1,\kappa }\\ \varpi_{2,1} & \varpi_{2,2} & \cdots & \varpi_{2,\kappa }\\ \vdots & \vdots & \ddots & \vdots \\ \varpi_{R,1} & \varpi_{R,2} & \cdots & \varpi_{R,\kappa } \end{array}\right].$$

The DMD modes are further reconstructed to generate feature vectors for the HMMs. Since the RSSI values in each set of observation samples are noisy and fluctuate, distinct frames are created by sliding windows over the succeeding number of RSSI samples without overlap. This process creates more informative depictions for the signal while preserving the temporal dynamics in consecutive windows. As a result, the noise effect is decreased, and RSSI signals are appropriately represented without losing spatial and temporal signal features.

Suppose the radio map of all fingerprints, $F_{j}\;j=1,2,\ldots ,R$ is to be divided into ι windows with *p* sampling points, given by:

(21) $$F_{\iota }={\left[\begin{array}{cccc} F_{1} & F_{2} & \cdots & F_{p} \end{array}\right]}^{T},\;\iota <j.$$

The feature vector for each window is obtained from (20) as:

(22) $$\Psi_{i}=\left[\begin{array}{cccc} {\left(\frac{1}{p}\sum_{q=1}^{p}\left|\varpi_{q}\right|\right)}_{1} & {\left(\frac{1}{p}\sum_{q=1}^{p}\left|\varpi_{q}\right|\right)}_{2} & \cdots & {\left(\frac{1}{p}\sum_{q=1}^{p}\left|\varpi_{q}\right|\right)}_{\kappa } \end{array}\right].$$

## 4. Hidden Markov Models

The HMM is a well-known probabilistic model that can be used to represent a sequence of discrete or continuous observations, which may be either time dependent or independent. The HMM, unlike traditional Markov models, assumes that physical states are unobservable (hidden) such that each observation is a stochastic relation of the hidden states. Therefore, the HMM is an effective modeling tool for classifying the sequence of RSSI observations (RSSI measurements) collected by the user device from different APs to improve the accuracy of mobile user positioning in a WiFi fingerprinting localization system. Let $S_{i},i=1,2,\cdots ,N$ be a set of *N* hidden states, each corresponding to an RP with coordinates in the localization area of interest, and $O_{j},j=1,2,\ldots ,R$ denotes the collection of *R* observed RSSI measurement vectors. The HMM is usually described by three major parameters, as follows:

1 Initial (prior) state probability, $\pi =\{\pi_{i}\},\;1\leq i\leq N$.
2 Transition probability matrix, $A=\{a_{ij}\}$. *A* is an N×N state matrix that shows the probability of transiting from state $s_{i}\in S$ to $s_{j}\in S$, given by:
  (23) $$a_{ij}=Pr\left(s_{j}|s_{i}\right),\;1\leq i,j\leq N.$$
3 Emission probability matrix, $B=\{b_{i}\left(o_{t}\in O\right)\}$. $b_{i}\left(o_{t}\right)$ represents the probability that an observation at time *t* is derived from a specific state $s_{i}$, given by:
  (24) $$b_{i}\left(o_{t}\right)=Pr\left(o_{t}|s_{i},\mu ,\sigma ,\omega \right),\;1\leq i\leq N,1\leq t\leq R,$$
  where μ,σ, and ω are the mean, covariance matrix, and mixture weight, respectively. These parameters specify the Gaussian emission distribution.

**Figure 1:**
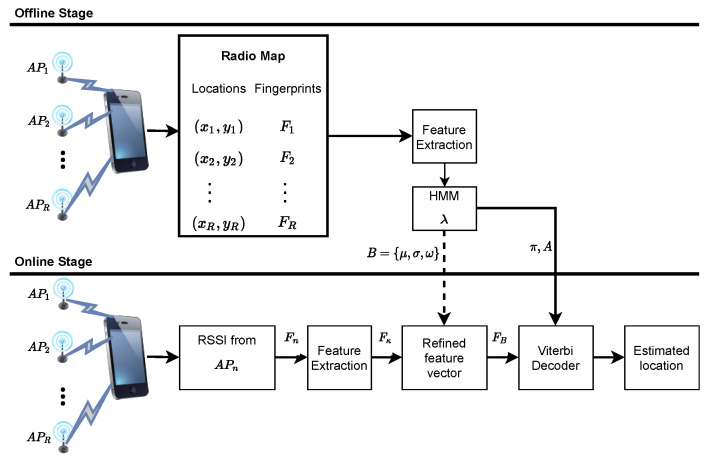
The system model for HMM-DMD indoor localization.

Figure 1 depicts the system design of the proposed WiFi fingerprinting localization for indoor environments. Suppose that π follows a uniform distribution similar to [5], and the fastest walking speed of a mobile user is 2 m/s. The location area can be divided into grid of reference points with equal grid spacing depending on the mobile user’s speed. Note that a grid may correspond to a room or portion of a corridor, indexed by a unique number (grid ID). Therefore, the localization problem becomes how to estimate the grid ID of the mobile user given the RSSI measurements collected from several APs at each RP.

In this study, an ergodic type HMM model λ={π,A,B}, containing *N* states is built for each grid of RPs such that all the states are connected to each other. This implies that transition is possible between any state; thus, the transition probabilities $a_{ij}$ are non-zero. Note that the probability of transiting between states only depends on the current state and not any other state. Each HMM node contains the emission probability distribution $b_{i}\left(o_{t}\right)$ for observing RSSI values on the grid of RPs. As such, a probabilistic road map for each grid of RPs is constructed by training the parameters of λ using the extracted feature vectors from Ω. As mentioned earlier, the effect of noise and multipath variation of the RSSI data from several APs in the grid of RPs is reduced by the proposed DMD feature extraction.

For a given sequence of observed feature vectors *O*, training the HMM requires computing the best parameters to maximize the probability of observing an RSSI measurement collected from different APs on a grid while also maximizing the joint probability of *O* and *S*, represented as:

(25) $$\begin{array}{cc} Pr(\lambda ,\lambda^{old})\; & =\sum_{s}Pr\left(S|O\lambda^{old}\right)\ln Pr\left(OS|\lambda \right). \end{array}$$

$\lambda^{old}$ is the current estimated parameters, λ is the parameters to be optimized, and $p(S|O,\lambda^{old})$ is the conditional probability distribution of *O*. Baum–Welch (BW) [23] is a well-known algorithm for iteratively refining the parameters of λ. The BW algorithm employs expectation maximization (EM) [24] to iteratively re-estimate the maximum likelihood of independent model parameters {λ=π,A,B}. At the start of the BW process, random values are initialized for the Gaussian emission distribution parameter B={μ,σ,ω}. However, this is assuming random start values depict the HMM positioning accuracy. Therefore, *k*-mean clustering is introduced to initialize the starting values for the parameters of *B*.

During the online stage, the acquired WiFi RSSI vector $F_{n}$ is compared to the trained HMM parameters, λ to estimate the unknown user location. Here, the extracted feature vector $F_{\kappa }$ from $F_{n}$ is first refined using parameters of *B* to obtain $F_{B}$. Thereafter, the π and *A* are compared with $F_{B}$ using the Viterbi algorithm [25] to obtain the best hidden state sequence corresponding to the unknown user current location. The extracted feature vector of the raw WiFi RSSI dataset is important to enhance the performance of the HMM and minimize the computational cost of the localization system. Therefore, DMD is implemented in this study for a proper representation of the fingerprints.

## 5. Experiment and Results

The proposed indoor localization system evaluation is based on a simulation model using CRAWDAD RSSI data collected from 802.11 APs at RPs on an office building floor at the University of Mannheim [26]. The test area is approximately 312 m${}^{2}$, having 14 APs and 612 grids of RPs with 0.5 m spacing in the area of interest. During the offline stage, 110 RSSI samples were collected at each RP, totaling 72,600 training samples. In addition, 110 samples were gathered at 83 randomly selected coordinates with the condition that they were within 4 RPs of the grid in the area of interest. Hence, a total of 9460 RSSI testing samples were collected for the online stage. The simulation and computation comparison were performed using MATLAB R2020b software on a MacBook Air (13 inch, 2017) with a 1.8 GHz Dual-Core Intel Core i5 processor, and 8 GB, 1600 MHz DDR3 memory size.

In this study, the localization distance errors (accuracy) and the cumulative distribution function (CDF) are employed as a performance metric. The localization distance error is determined by the Euclidean distance, which measures the distance between the model’s approximated location and the real coordinate. In the experiments, the proposed (HMM-DMD) localization approach is compared to the conventional algorithms such as WKNN [1], random forest (RF) [27], and naive Bayes (NB) [28] for indoor WiFi fingerprinting localization.

The parameters of each algorithm are chosen as follows. For WKNN, values of *k* were selected to avoid ties so that each neighbor RSSI vector has a weight based on its Euclidean distance from the test RSSI vector. Similar to [1], k=10, corresponding to the number of common RPs ($\bar{k}=7$) was used to recompute the weights of selected RPs based on physical and spatial distances of RPs in each grid. RF is a decision tree ensemble-based ML algorithm, which produces high accuracy in a variety of applications. When generating the forest, the number of iterations is fixed at 100, and all attributes are randomly investigated. For the NB algorithm, default parameters are used since it utilizes Bayesian statistics. The dimension of the extracted feature is determined by the SVD target rank κ, which is a crucial parameter to consider in the proposed DMD. To reduce computing cost in the HMM process, the value of *r* should be kept as small as possible without losing relevant information. Here, the most suitable κ is obtained based on simulation.

### 5.1. Performance Analysis for Different DMD Ranks, κ

The mean localization error, $\hat{V}$, and percentage of improvement of the proposed HMM-DMD indoor localization system are first examined by varying the ranks as κ. Note that different HMMs are developed for each room $R_{1}$ and $R_{2}$ in the localization area of interest. Table 1 depicts the performance of the HMM-DMD at κ=5,6,7,8,9, and 10 with respect to the RSSI sample sizes *p*. For all sample sizes, the mean localization errors of the proposed HMM-DMD localization algorithm decrease while the performance of the algorithm improves as the rank increases from κ=5 to 7. Moreover, with a further increase in κ from 7 to 10, the mean localization error and percentage of improvements remain insignificant. Hence, increasing κ beyond 7 does not necessarily improve the performance of the HMMs, but it does raise the computational cost.

### 5.2. Performance Comparison of Different Training Sample Sizes, p

**Figure 2:**
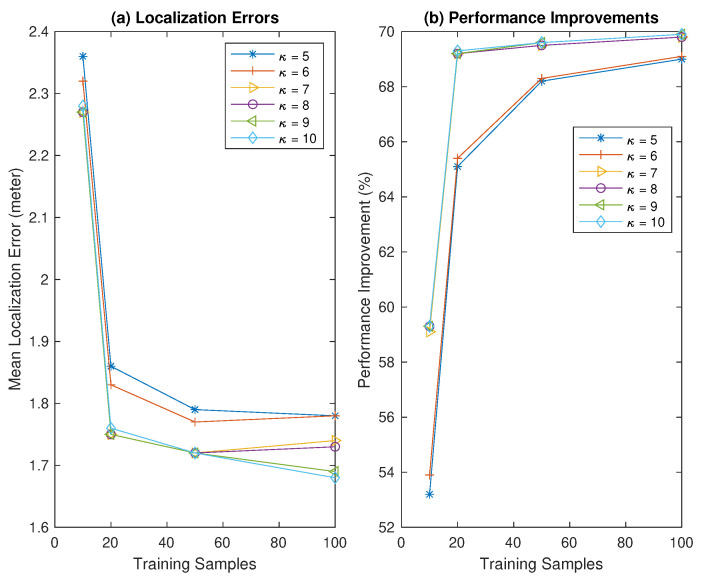
Performance of proposed HMM-DMD with different κ at varying training sample sizes *p*.

The impact of utilizing different training samples obtained at each RP on the mean localization error and percentage of improvements of the proposed HMM-DMD with varying κ is shown in Figure 2. The results are consistent with Table 1. Figure 2a illustrates that the localization error significantly decreases by increasing the size of training samples, *p* from 10 to 20 for all values of κ. Moreover, increasing *p* from 20 to 50 yields a localization error below 7 cm. Further increasing *p* from 50 to 100 only saturates the localization error, except for κ=9 and 10.

Similarly, Figure 2b depicts that the HMM-DMD achieves significant performance improvements by employing 20 samples compared to p=10 for all values of κ. Additionally, using training samples of 50 and 100, the performance of the HMM-DMD improves by 68.2% and 69.0% with κ=5, and 68.1% and 69.1% with κ=6, respectively. Nevertheless, the result shows that the performance improvements of the HMM-DMD with κ=7,8,9, and 10 remain constant by using additional 50, and 100 samples. Therefore, 20 training samples with κ=7 are selected for the HMM-DMD localization algorithm in this study since they provide an enhanced tradeoff between the localization error and complexity cost.

### 5.3. Performance Comparison of the Proposed HMM-DMD with State-of-the-Art Methods

**Figure 3:**
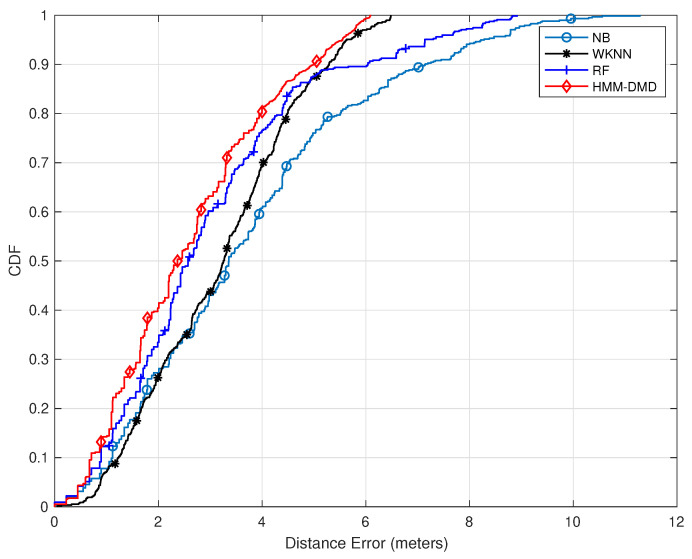
Comparison of the proposed HMM-DMD with conventional ML techniques (κ=7,p=20).

In this section, the proposed HMM-DMD localization technique at κ=7 and p=20 is compared with the well-known WKNN, RF, and NB. Figure 3 depicts the CDF of localization errors for all methods. The proposed HMM-DMD algorithm exhibits an enhanced performance in comparison with each of the ML techniques. The HMM-DMD yields a performance improvement of 40% within 2 m compared to RF, WKNN, and NB with performance improvements of 30.3%, 25% and 27.2%, respectively. This is due to the DMD feature extraction, which eliminates the noise effects and decomposes the huge RSSI features to relevant spatial–temporal modes without losing relevant information.

**Figure 4:**
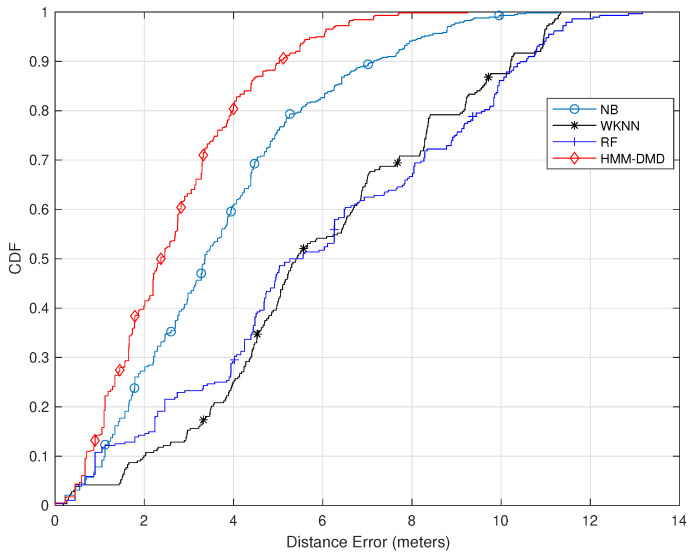
Comparison of the proposed HMM-DMD with conventional ML techniques (κ=9,p=100).

Figure 4 further shows the result of utilizing 100 training samples and feature dimension κ=9. The figure clearly indicates that within 2 m, the HMM-DMD exhibits a performance improvement of 12.7%, 38.6%, and 39% over the NB, RF, and WKNN, respectively. Furthermore, at 4 m, the proposed algorithm outperforms the NB, RF, and WKNN by 20%, 50%, and 54.7%, respectively. Additionally, the HMM-DMD is able to attain improvements of 12%, 43.7%, and 40% over the NB, RF, and WKNN, respectively, within 6 m.

To further emphasize the performance of the proposed HMM-DMD, the overall accuracy and the processing time of the proposed HMM-DMD and the conventional approaches with κ=7 and p=100 is presented in Table 2. The HMM-DMD algorithm yields an overall accuracy of 94.65% compared to the NB, RF, and WKNN with overall accuracy of 91.68%, 89.37%, and 87.61%, respectively. In comparison with the state-of-the-art algorithms, the high accuracy of the proposed algorithm comes at the cost of processing time. The main time-consuming process is the creation of a fingerprint database at selected reference points in an area of interest. The proposed HMM-DMD has a training time of 3.0067, which is relatively low when compared to the time it takes to develop a fingerprint database. Additionally, the WiFi RSSI will be slowly altered since indoor settings are rarely changed. Therefore, the fingerprinting database would only be updated after a long period of time if there was a significant decrease in localization performance. The testing time of the proposed algorithm for 100 testing samples is 0.1600 s. This implies that each test sample requires only 0.00016 s of processing time. Hence, the proposed HMM-DMD algorithm is suitable for real-time WiFi fingerprinting indoor localization.

## 6. Conclusions

This article presented a DMD approach for reducing the dimensionality of WiFi RSSI features to be employed in the HMM to achieve efficient indoor localization. The DMD decomposes raw WiFi signals into spatial–temporal modes, which were converted into HMM-ready feature vectors. This process reduces the effect of noise and varying visibility of the access points. To increase the localization performance, the HMM was used to train high-level reconstructions from the retrieved features and classify the sequence of observations. The accuracy and performance improvements of the proposed localization model were verified using simulations. The effects of varying the rank of DMD and using different numbers of training samples were investigated on localization errors. This assisted in selecting the best parameters for reducing the computational cost and obtaining enhanced accuracy. Additionally, the results of comparing the proposed HMM-DMD localization algorithm to the state-of-the-art approaches showed a significant performance improvement, high accuracy, and reasonable processing time, thus making the proposed algorithm appropriate for real-time WiFi fingerprinting indoor localization. Future research should consider the use of additional sensors for multifloor identification, as floor identification based on only WiFi may exhibit poor performance.

## Figures and Tables

**Table 1:** Localization errors and performance improvement of HMM-DMD for different κ.

*p* (Samples)	Criterion	κ=5	κ=6	κ=7	κ=8	κ=9	κ=10
10	$\hat{V}$ (m)	2.36	2.32	2.27	2.27	2.27	2.28
	Improvement (%)	53.2	53.9	59.1	59.3	59.3	59.3
20	$\hat{V}$ (m)	1.86	1.83	1.75	1.75	1.75	1.76
	Improvement (%)	65.1	65.4	69.2	69.2	69.2	69.3
50	$\hat{V}$ (m)	1.79	1.77	1.71	1.71	1.71	1.70
	Improvement (%)	68.2	68.3	69.5	69.5	69.6	69.6
100	$\hat{V}$ (m)	1.78	1.77	1.69	1.69	1.69	1.68
	Improvement (%)	69.0	69.1	69.8	69.8	69.9	69.9

**Table 2:** Accuracy and processing time comparison for different localization methods.

Methods	Accuracy (%)	Time (s)	
		Testing	Training
WKNN	87.61	4.9856	0.0019
RF	89.37	0.2240	3.6905
NB	91.68	0.0177	1.1217
HMM-DMD	94.65	0.1600	3.0067

## Data Availability

This study analyzed publicly available datasets. The data are available at https://crawdad.org/mannheim/compass/20080411/802.11 (accessed on 3 May 2021).
